# Deletion of Nrf2 induced severe oxidative stress and apoptosis in mice model of diabetic bladder dysfunction

**DOI:** 10.1007/s11255-024-04064-y

**Published:** 2024-05-21

**Authors:** Lei Wang, Weiaho Sun, Guanyu Ren, Yi Sun, Cheng Xu, Qixiang Song, Xinhui Zhang, Chenghua Yang, Zhiyong Liu

**Affiliations:** 1 Department of Urology Surgery, Changhai Hospital, Naval Military Medical University, Shanghai, 200433 China; 2 Department of Urology Surgery, Renji Hospital, ShangHai JiaoTong University, Shanghai, 200433 China; 3 Department of Pharmacology, School of Pharmacy, China Pharmaceutical University, Nanjing, 210009 China

**Keywords:** Type 2 diabetes mellitus, Nrf2, Diabetic bladder dysfunction, Oxidative stress, Apoptosis

## Abstract

The nuclear factor erythroid 2-related factor 2 (Nrf2) pathway has been confirmed as a therapeutic target for type 2 diabetes mellitus (T2DM), however few studies revealed its effect in diabetic bladder dysfunction (DBD). Herein, we reported a Nrf2 deletion diabetic mouse model induced by 8-week high-fat diet feeding combined with streptozocin (STZ) injection in Nrf2 knockout mice. Besides, wild-type mice (WT) were used as control group, wild-type mice with high-fat diet feeding and STZ injection as diabetic group (WT-T2DM), and Nrf2 knockout mice as Nrf2 deletion group (KO). The pathophysiological indexes and bladder morphology showed typical pathological features of diabetic bladder dysfunction in Nrf2 knockout diabetic mouse mice (KO-T2DM). ELISA results showed that advanced glycation end products (AGEs), ROS and malondialdehyde (MDA) levels in bladder was were up-regulated in both WT-T2DM and KO-T2DM group, while superoxide dismutase (SOD) and glutathione (GSH) levels decreased in these two groups. Compared with WT-T2DM group, western blot analysis of the bladder showed down-regulated expression of NQO1 and HO-1 in KO-T2DM group. However, apoptosis, marked by Caspase3 and bax/bcl-2 ratio, was increased in KO-T2DM group. Neurotrophic factor (NGF) was significantly decreased in DBD model, and even much lower in KO-T2DM group. Collectively, our findings demonstrated that deletion of Nrf2 lead to severe oxidative stress, apoptosis, and lower level of neurotrophic factor, and provided the first set of experimental evidence, in a mouse model, to support Nrf2 as a promising target for DBD.

## Introduction

Diabetes mellitus (DM) is one of the most common endocrine system diseases in the world. According to new published International Diabetes Federation (IDF) diabetes atlas, the global diabetes prevalence in 20–79-year-old in 2021 was estimated to be 10.5% (536.6 million people), which will rise to 12.2% (783.2 million) in 2045 [1]. More than half of diabetic patients with poor blood glucose control suffer from the secondary onset of diabetes bladder dysfunction (DBD) [2, 3]. DBD involves various symptoms, including problems of urinary storage period, such as overactive bladder and acute urinary incontinence, as well as urination problems, such as bladder emptying disorder, urinary retention, and urinary incontinence [4].

DBD has very complex etiology and pathogenesis. Now, the specific pathogenesis of DBD is still unclear. Current research results show that oxidative stress plays a significant role in the pathogenesis of DBD, with a possible link between functional changes in urothelium, muscle and the corresponding innervations [5]. Nuclear factor erythroid 2-related factor 2 (Nrf2) is a vital regulatory molecule against oxidative stress. As a transcription factor, Nrf2 can combine with antioxidant response element (ARE) to form Nrf2-ARE signaling pathway and regulate the expression of downstream antioxidant protein molecules [6]. The researches of Nrf2 and DBD are still in its infancy, and the specific mechanism is still unknown [7]. The paper aims to explore the role and mechanism of Nrf2 in DBD.

## Methods

### Establishment of animal model

All animal experiments were performed in accordance with the the Guide for the Care and Use of Laboratory Animals published by the US National Institutes of Health and approved by Institutional Committee of Health Guide and Care, China Pharmaceutical University. Male C57BL/6 J mice (8 weeks old) were purchased from Yangzhou University Medical Center (Yangzhou, China). Nrf2 knockout mice (B6.129X1-*Nfe2l2*^*tm1Ywk*^*/J*) were purchased from the Jackson Laboratory (stock No. 017009). Mice were housed under the standard laboratory environmental conditions (temperature 20 ± 2 °C, humidity 50%, and regular light/dark cycle (12 h/12 h)), with free food and water access. The animals were adapted to tested conditions for at least 1 week.

Type 2 diabetes mellitus was induced by feeding with high fat die (HFD, 60% energy from fat). After 12 weeks, HFD animals were received i.p. injection of streptozotocin (STZ, 60 mg/kg in citrate buffer, 10 mmol/l, pH 4.5 on 5 consecutive days) after 4–5 h fasting. Fasting serum glucose (FSG) was checked after 7 days using a glucometer (Contour next EZ, Bayer, ON, Canada) from the tail. FSG exceeded 12 mmol/l was considered diabetic. Body weight was recorded after the model completing. Animals were sacrificed by CO2 gas inhalation and bled rapidly by cutting the carotid arteries to collect the blood sample and bladder tissue.

### Immunohistochemistry and histology

Organs were fixed with formalin and embedded in paraffin. 5 μm paraffin sections were cut and stained with Masson's Trichrome Stain Kit (Solarbio, Beijing, China).

The Sects. (10 μm) were deparaffinized in xylene and rehydrated in a graded series of ethanols (100%, 95%, 80%, and 75%) for 5 min each, followed by antigen recovery. The sections were then incubated in 3% H2O2 at 37 °C for 15 min, washed with PBS, incubated with goat serum for 30 min at 37 °C, and then incubated with primary anti-Nrf2 antibody (1:100, Abcam, Cat# ab137550) at 4 °C overnight. The next day, the sections were incubated with secondary goat anti-rabbit antibody (ZsBio, Beijing, China) for 45 min at 37 °C followed by an ABC working solution (ZsBio) for 25 min at 37 °C and incubated with 3,3-diaminobenzidine (ZsBio). Ten visual fields were randomly chosen for each sample under a LEICA DM600B automatic microscope (Leica Microsystems, Heidelberg GmbH, Germany). Nrf2 expressions were quantified using Image-Pro Plus 6.0 (Media Cybernetics), and the mean density value (integrated optical density divided by the relevant area) was calculated for each visual field.

### Biochemical assay

Plasma oxidative and anti-oxidative factors in bladder, including advanced glycation end products (AGEs), reactive oxygen species (ROS), superoxide dismutase (SOD), malondialdehyde (MDA), and glutathione (GSH), were assayed using kits obtained from Jiancheng Bioengineering Co., Nanjing, Jiangsu, China, following the instructions.

### Western blotting

Bladder tissues were placed in RIPA lysis buffer, containing a protease inhibitor cocktail (Roche Diagnostics, Indianapolis, IN, USA). Cytoplasmic and nuclear proteins were extracted from fresh tissues, following the kit instructions (Invent Biotechnologies. Inc., sc-003). Protein samples (30 μg) were separated with SDS-PAGE and transferred onto polyvinylidene difluoride (PVDF) membranes (Amersham Biosciences, Piscataway, NJ, USA). The membrane was blocked with blocking buffer (TBS, 0.1% Tween-20, 5% non-fat milk or 2% BSA), for 2 h at room temperature, and incubated with primary antibodies against Nrf2 (1:1000, Abcam, Cat# ab137550), NO-1(1:1000, Abcam, Cat# ab13248), NQO1 (1:1000, Abcam, Cat# ab34174), Caspase3 (1:1000, Cell Signaling Technology, Cat# 9662), Bax (1:1000, Cell Signaling Technology, Cat# 41,162), Bcl-2 (1:1000, Cell Signaling Technology, Cat# 15,071), pro-NGF (1:1000, Abcam, Cat# ab52918), NGF (1:1000, Cell Signaling Technology, Cat# 2046), lamin B (1:1000, Cell Signaling Technology, Cat# 13,435) and β-actin (1:5000, Santa Cruz, Cat# sc-8432), at 4 °C overnight. Goat anti-rabbit IgG horseradish peroxidase (1:10,000, Abcam, Cat# ab6721) or goat anti-mouse IgG horseradish peroxidase (1:5000, Abcam, Cat# ab6789) were incubated for 60 min at room temperature the next day, respectively. The blots were developed with ECL kit (Applygen Technologies Inc, Beijing, China) and finally exposed to X-ray films. Relative protein expression levels were quantified by optical density analysis (Quantity-One software, Bio Rad Gel Doc 1000, Milan, Italy) and normalized to β-actin.

### Data analysis

All the data were obtained from at least three independent experiments, and the representative results are presented. All results are expressed as mean ± SD. Statistical analysis was conducted using Prism 7 software (GraphPad Software). Statistical significance was evaluated using the unpaired two-tailed Student’s t-test or one-way ANOVA with post-hoc test among more than two groups. The significant difference was considered if p < 0.05 (n ≥ 3).

## Result

### Effect of Nrf2 deletion on the physiology condition of type 2 diabetes mice

**Fig. 1:**
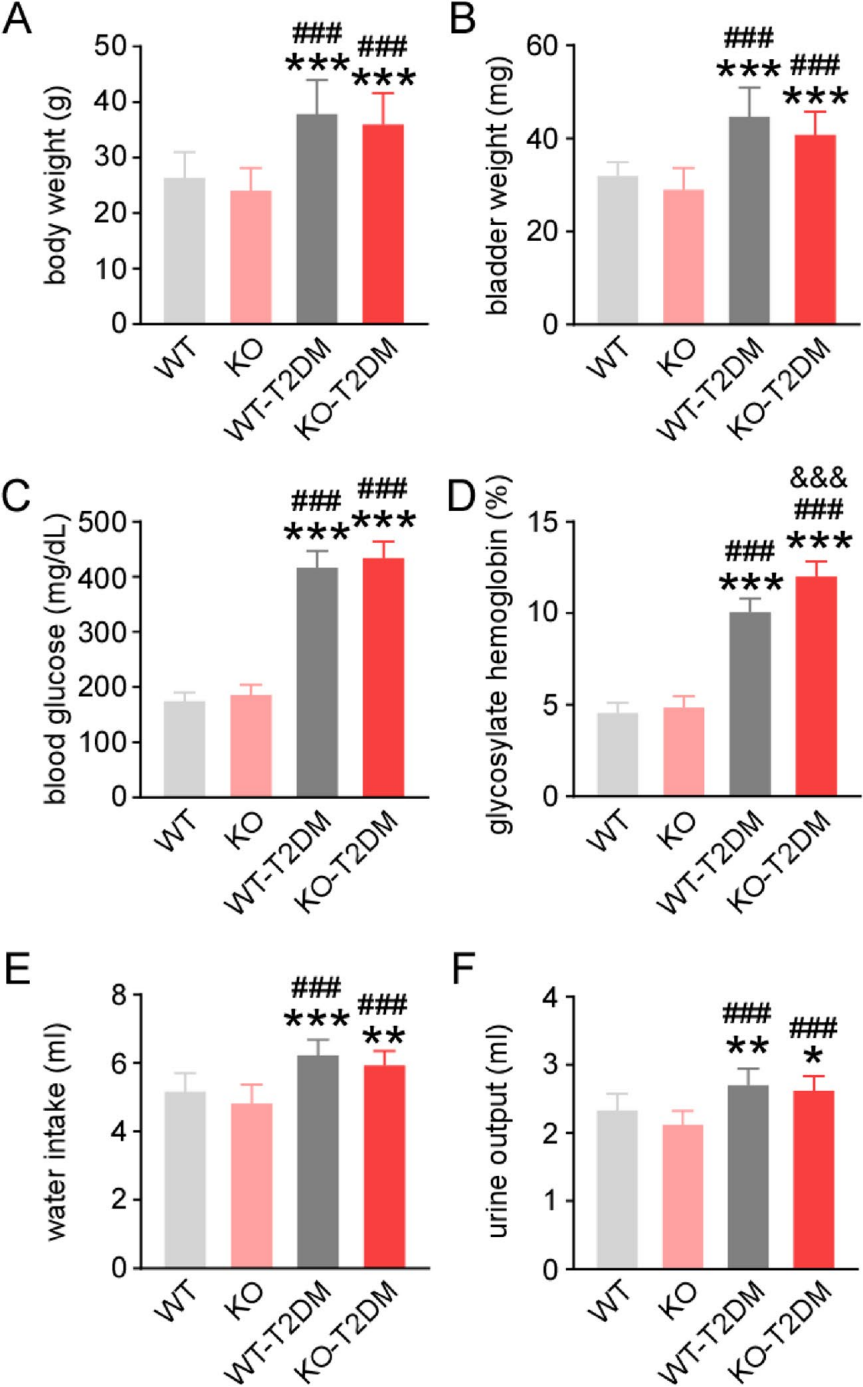
The physiology condition of mice after modeling. The changes of bladder weight (**A**), bladder weight (**B**), blood glucose (**C**), glycosylated hemoglobin level (**D**), the volume of daily water intake (**E**), and the volume of urine output (**F**). WT, C57BL/6 mouse group with normal diet; KO, Nrf2 knockout mouse with normal diet; WT-T2DM, C57BL/6 mouse given high fat diet and STZ injection; KO-T2DM, Nrf2 knockout mouse given high fat diet and STZ injection. Data are reported as Mean ± SEM, n = 10. *P < 0.05, *** P < 0.001 vs WT. ### P < 0.001 vs KO. &&& P < 0.001 vs WT-T2DM

There were used *Nrf2* knockout (KO) mice to confirm the critical role of Nrf2 in diabetic bladder dysfunction development. Wild type mice of type 2 diabetes mellitus (WT-T2DM) and *Nrf2* knockout mice of type 2 diabetes mellitus (KO-T2DM) were induced by feeding with high fat diet for 12 weeks and later injected intraperitoneally with STZ. Wild type mice with normal diet (WT) were used as the control group. The results showed that WT and KO mice have comparable body weight, bladder weight, blood glucose, glycosylated hemoglobin value, water intake, and urine output, indicating that deletion of Nrf2 has little effect on the growth and basic physiology of mice (Fig. 1A to 1F). With high fat diet and STZ injection, the body weight, blood glucose, and glycosylated hemoglobin value were increased in both WT-T2DM and KO-T2DM groups than WT and KO groups. Besides, WT-T2DM and KO-T2DM groups had significantly higher daily water intake and urine output than WT and KO group, which showed as the typical symptoms of T2DM as in clinic. All these suggested successful establishment of T2DM model.

However, there seemed no difference of the body weight, the blood glucose, daily water intake and urine output, between WT-T2DM and KO-T2DM groups. Only the glycosylated hemoglobin value was higher in KO-T2DM mice than WT-T2DM mice, suggesting that Nrf2 deletion maybe caused long-term changes of blood glucose and more severe diabetic complications.

### Effect of Nrf2 deletion on the bladder morphology of type 2 diabetes mice

**Fig. 2:**
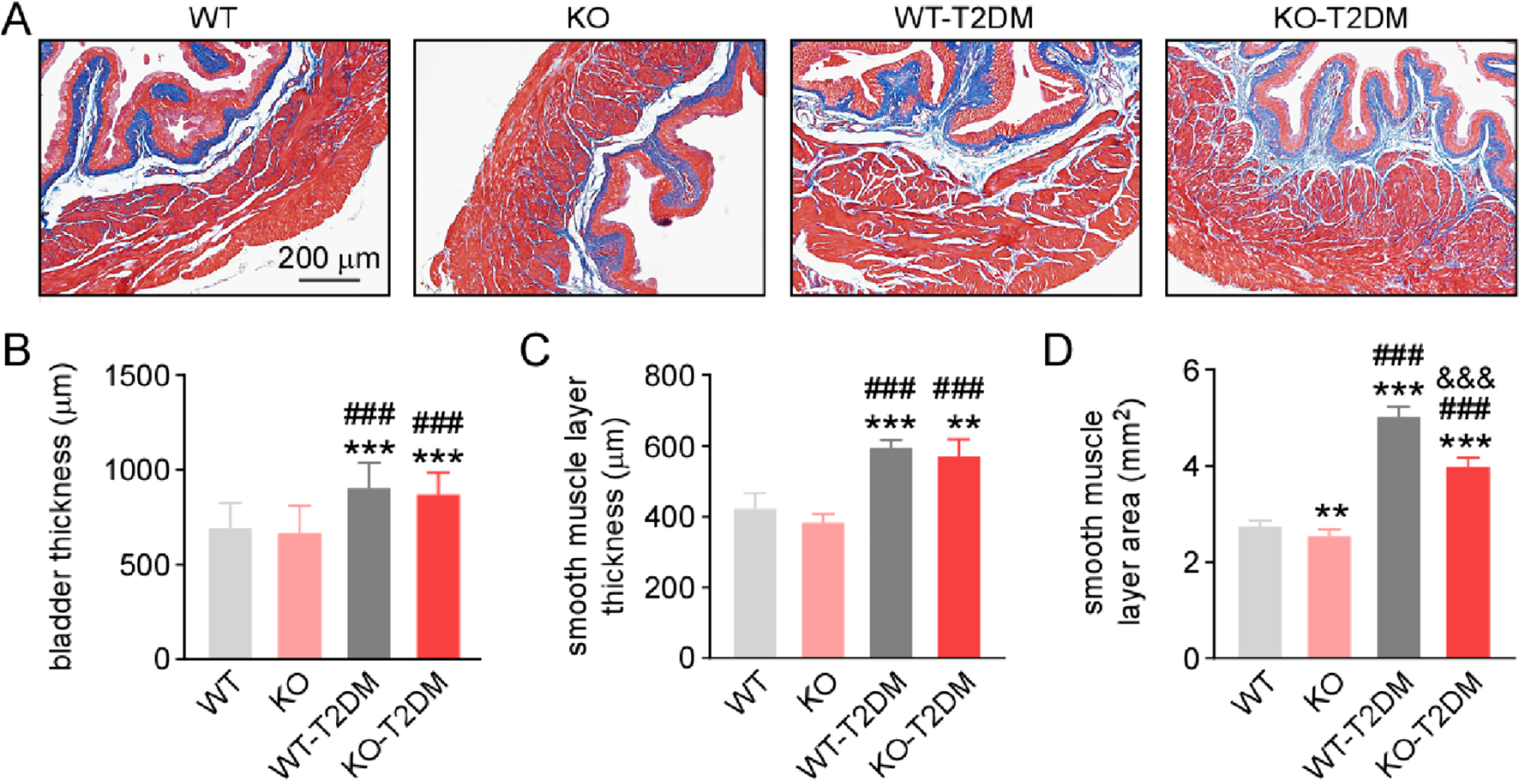
Effect of deletion of Nrf2 on the histology of mice bladders. **A **Masson staining images of mouse bladder tissues. Bar = 200 μm. **B** The statistical graphs of bladder wall thickness. **C** The statistical graphs of the bladder smooth muscle layer thickness. **D** The statistical graphs of the area of bladder smooth muscle layer. Data are reported as Mean ± SEM, n = 10. **P < 0.01, *** P < 0.001 vs WT. ### P < 0.001 vs KO. &&& P < 0.001 vs WT-T2DM

To further confirm whether Nrf2 deletion resulted in bladder structure changes, the Masson staining was performed, as well as the thickness and the area of the bladder were measured to explore the bladder morphology. As stained blue, there was a growing amount of the collagen in WT-T2DM mice and KO-T2DM mice (Fig. 2A). And as counterstained red, the amount of muscle reduced in WT-T2DM mice and KO-T2DM mice (Fig. 2A). The results indicated a progressive disruption of the detrusor smooth muscle layer of the bladder wall and increased deposits of collagen fibers in musculature of T2DM mice. In addition, Nrf2 deletion aggravated collagen deposition and hypertrophy of the muscles in the bladder, suggesting the bladder dysfunction in T2DM mice and Nrf2 knockout mice.

The results showed no significant difference between the WT group and KO group in terms of the bladder wall thickness and the smooth muscle layer thickness (Fig. 2B). Both the WT-T2DM mice and the KO-T2DM mice had thicker bladder wall than WT mice or KO mice (Fig. 2B). Especially, the thickness of the smooth muscle layer changed in a similar way consistently with the bladder wall among the four groups (Fig. 2C). Although the bladder became thickened, the area of the smooth muscle layer became smaller in mice of high blood glucose than those of normal blood glucose (Fig. 2D). Moreover, Nrf2 knockout alone caused reduced area of the smooth muscle layer (Fig. 2D). All these illustrated that deletion of Nrf2 reduced the function of the bladder smooth muscle layer, which may further result in compensatory hyperplasia.

### Effect of Nrf2 deletion on the oxidative pathway in type 2 diabetes mice

**Fig. 3:**
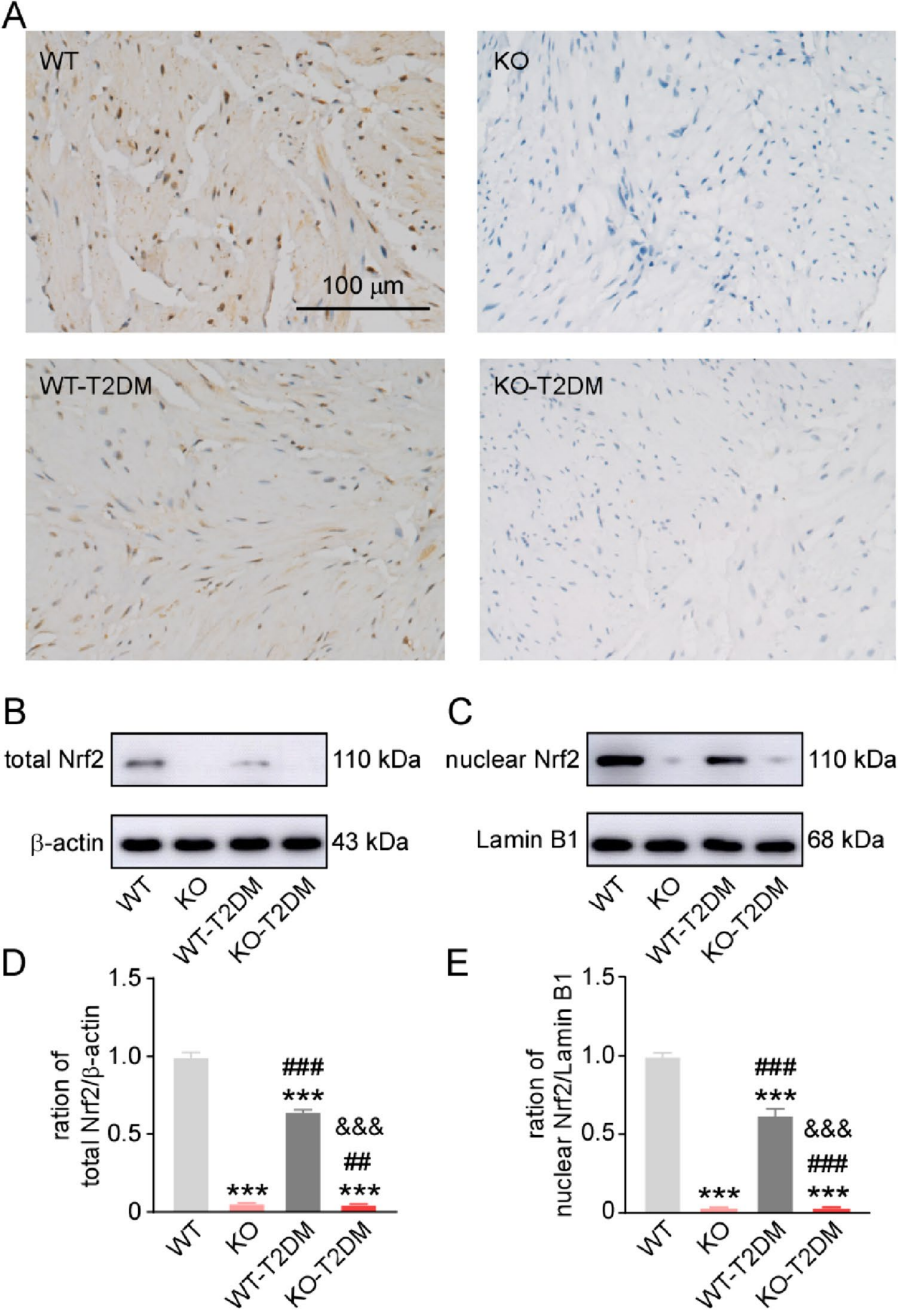
The expression level of Nrf2 in the bladder tissues. **A** The immunohistochemical images of Nrf2 in the bladder tissues of the four groups. **B**, **D** The typical western image and statistical graph of Nrf2 in the whole cell of the bladder tissue. **C**,**E** The typical western image and statistical graph of Nrf2 in the nucleus of the bladder tissue. Data are reported as Mean ± SEM, n = 5. *** P < 0.001 vs WT. ### P < 0.001 vs KO. &&& P < 0.001 vs WT-T2DM

The results of immunohistochemical staining (Fig. 3A) and western blot (Fig. 3B-E) showed that high fat diet combined with STZ injection reduced the expression level of both total Nrf2 and the nuclear Nrf2 in WT mice, suggesting more severe oxidative stress in T2DM mice.

**Fig. 4:**
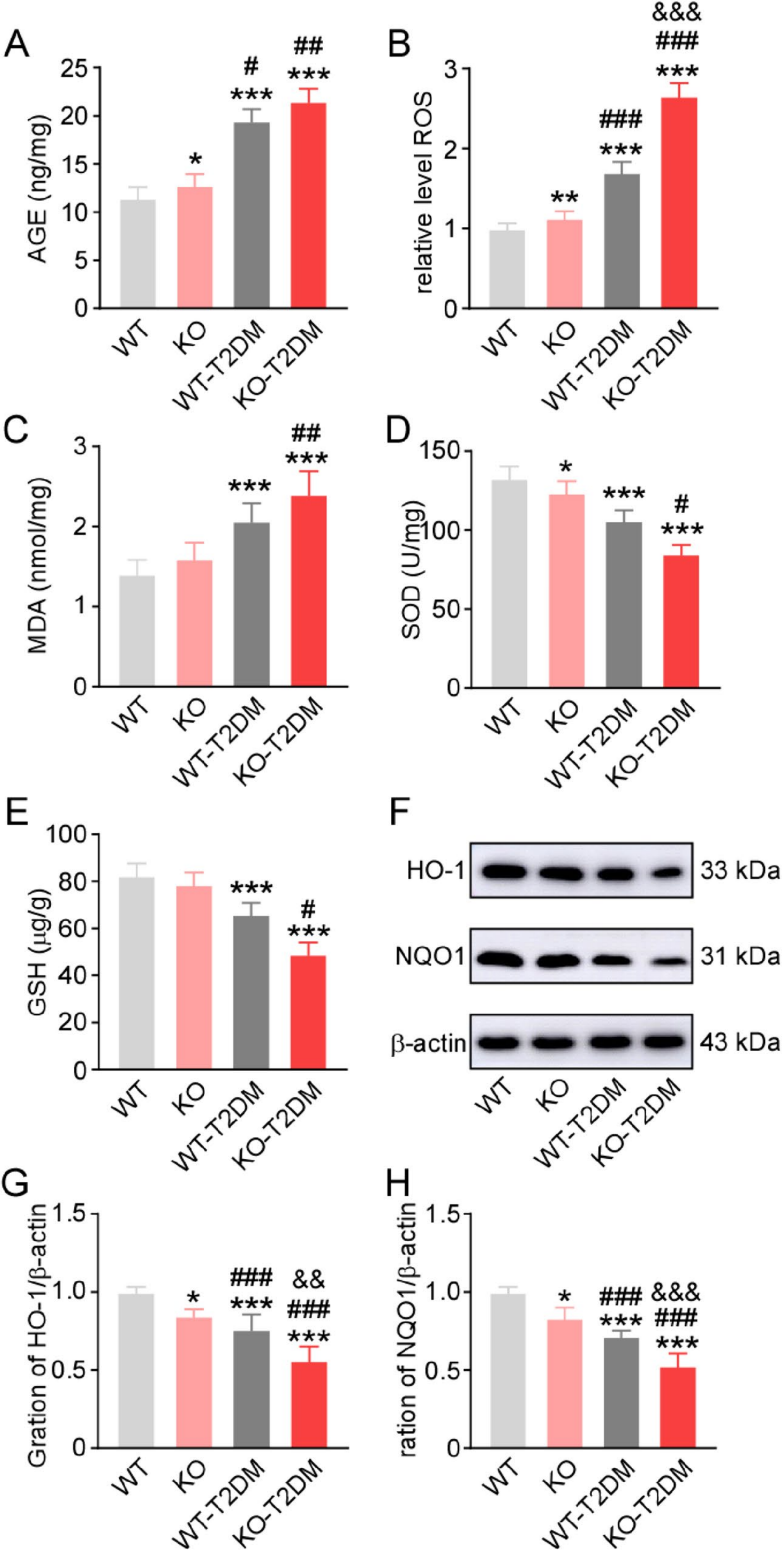
The expression level of related oxidative stress molecules and antioxidant stress molecules in the bladder tissue. The level of AGEs (**A**), ROS (**B**), MDA (**C**), SOD (**D**), and GSH (**E**) in bladder tissue of the four groups. The typical western blotting images (**F**) and the statistical graphs of NQO1 (**G**) and HO-1 (**H**). Data are reported as Mean ± SEM, n = 5. * P < 0.05, **P < 0.01, *** P < 0.001 vs WT. # P < 0.05, ## P < 0.01, ### P < 0.001 vs KO. && P < 0.01, &&& P < 0.001 vs WT-T2DM

Advanced glycation end products (AGEs, Fig. 4A), the typical oxidative products of glucose in the blood, increased in KO, WT-T2DM and KO-T2DM groups.

Nrf2 is a transcription factor that controls the basal and inducible expressions of many antioxidant and detoxification enzymes. Several down-stream oxidative-related molecules were tested to reflect the redox state, including reactive oxygen species (ROS, Fig. 4B), dismutase malondialdehyde (MDA, Fig. 4C) superoxide (SOD, Fig. 4D), and glutathione (GSH, Fig. 4E). The results showed that the anti-oxidative factors (SOD and GSH) were reduced in the bladder of the T2DM model. MDA, an oxidative stress biomarker, increased in T2DM mice. All these consistent with the changes of AGEs levels in four groups.

Besides, the expression levels of Nrf2-targeted antioxidant enzymes, including HO-1 (Fig. 4F, 4G) and NAD(P)H quinone oxidoreductase-1 (NQO1, Fig. 4F, 4H) decreased in Nrf2 knockout mice and T2DM mice, suggesting that Nrf2 deletion lead to the down-regulation expression of its downstream dependent genes. These results demonstrated that Nrf2 deletion aggravated oxidative stress in T2DM mice bladder.

### Effect of Nrf2 deletion on the apoptosis pathway in type 2 diabetes mice

**Fig. 5:**
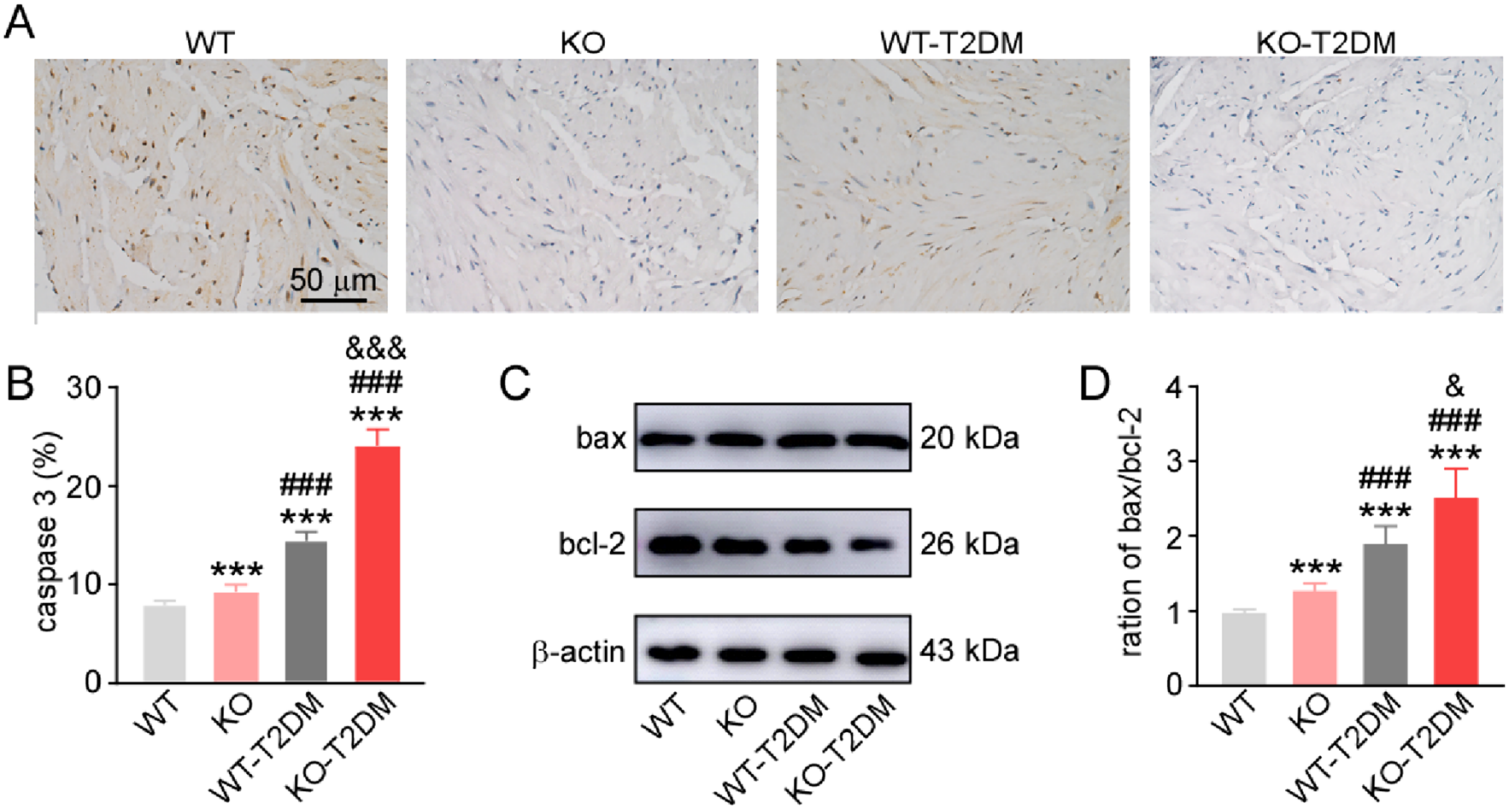
The expression levels of apoptosis pathway molecules. **A** The immunohistochemical staining images of caspase 3. **B** The statistical graphs of the expression level of caspase 3. **C** The typical western blotting images of bax and bcl-2. **D** The ration of the expression of bax to bcl-2. Data are reported as Mean ± SEM, n = 5. *** P < 0.001 vs WT. ### P < 0.001 vs KO. & P < 0.05 vs WT-T2DM

Several studies already clarified that oxidative stress induced apoptosis in different cell models or animal models [8–10].Here some classic molecules were tested to identify the effect of Nrf2 deletion on the apoptosis pathway in type 2 diabetes mice. There was an increase of the expression level of caspase 3, an apoptosis-related molecule, in the bladder tissues of KO group compared with WT group (Fig. 5A, B). The expression levels of caspase 3 in the bladder tissues of WT-T2DM mice and KO-T2DM mice were obviously increased, and Nrf2 deletion even up-regulated the level of caspase-3 (Fig. 5A, B).

In addition, there were nearly the same expression changes of bax/bcl-2 ratio in these four groups. With T2DM modeling, the ratio of bax to bcl-2, standing for cellular apoptosis condition, was increased in WT mice or KO mice (Fig. 5C, D). Knockout of Nrf2 caused the increased ratio of bax to bcl-2 in both KO mice and KO-T2DM mice (Fig. 5C, D). All these suggested that apoptosis occurred in type 2 diabetic mice with normal or high blood glucose as well as Nrf2 deletion aggravated apoptosis, which may contributed to the bladder dysfunction.

### Effect of Nrf2 deletion on the expression level of nerve growth factor in type 2 diabetes mice

**Fig. 6:**
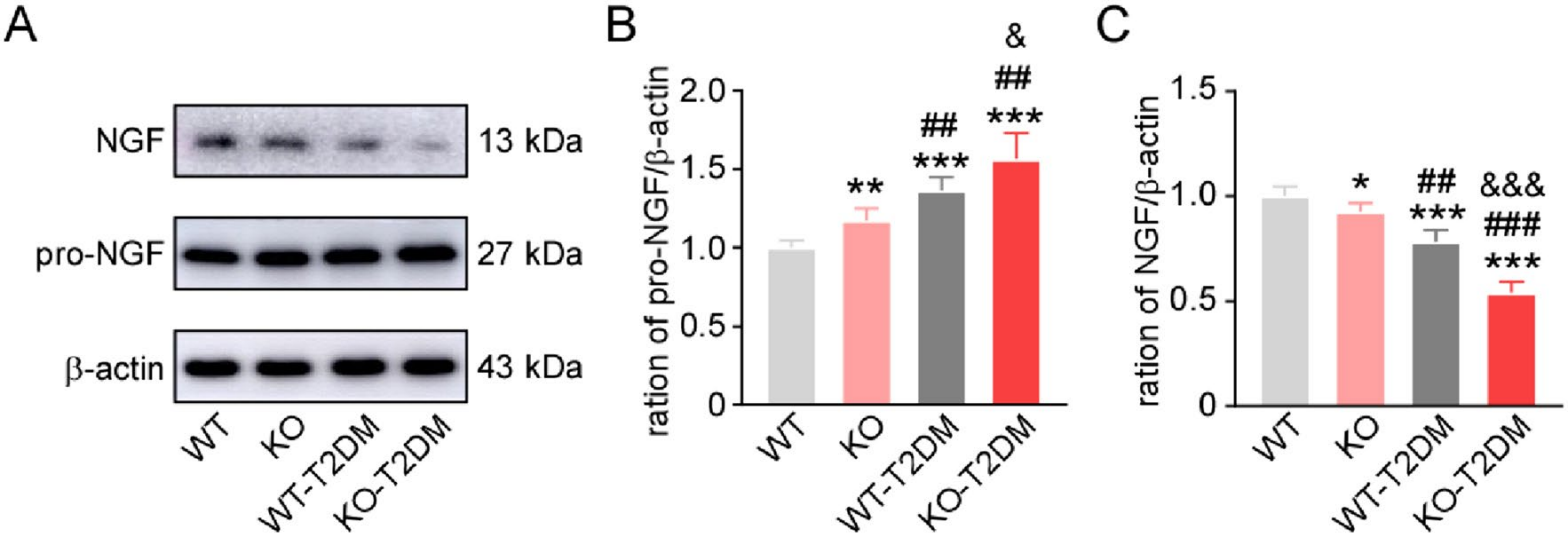
Effect of deletion of Nrf2 on the expression level of NGF and Pro-NGF. (**A**)The typical western blot images of NGF and pro-NGF. The statistical graphs of expression levels of NGF (**B**) and pro-NGF (**C**). Data are reported as Mean ± SEM, n = 5. * P < 0.05, **P < 0.01, *** P < 0.001 vs WT. ## P < 0.01, ### P < 0.001 vs KO. & P < 0.05, &&& P < 0.001 vs WT-T2DM

As the nerve growth factor (NGF) was associated with overactive bladder symptoms and other bladder dysfunction, there was a need to analyze the expression levels of NGF and pro-NGF in the four groups [11, 12]. Compared with WT mice, the expression level of pro-NGF was up-regulated in KO mice (Fig. 6A, B), while the expression level of NGF was down-regulated in KO mice (Fig. 6A, C). What’s more, the relative expression level of NGF in KO-T2DM group was significantly lower than that in KO group, and the relative expression level of NGF in WT-T2DM group was significantly lower than that in WT group.

## Discussion

As the most common complication of DM, DBD presents many symptoms such as overactive bladder, bladder emptying disorder and urinary retention, which seriously affects patients’ quality of life [13]. In the study, the mechanism of Nrf2 in the pathogenesis of DBD is further explored by establishing T2DM mice model and Nrf2 gene knockout mouse with T2DM. Different groups of mouse models are successfully constructed, including WT-T2DM group and KO-T2DM group. According to the research findings, there is no significant difference between KO group and WT group in terms of physiological indexes and bladder morphology. Therefore, the simple knockout of Nrf2 gene does not change the physiological indexes such as body weight, blood sugar and bladder thickness of the mice, nor obvious injury to the bladder tissue cells of mice (Fig. 1, Fig. 2). In the study, the molecular expression level of Nrf2 in the bladder tissues of the four groups is also detected through western blot. It is found that the expression level of total Nrf2 and intranuclear Nrf2 in the bladder tissues of the mice in the T2DM group is obviously lower than that in the WT group (Fig. 3). Nrf2 is decreased in the process of T2DM. The expression level of caspase3 and bax/bcl-2 in bladder tissue is higher than that in the other three groups, showing that KO-T2 DM group has more severe bladder tissue injury and apoptosis than WT-T2DM group (Fig. 5).

Oxidative stress can induce the apoptosis of bladder smooth muscle, and can also affect the activity of nerve cells by interfering the synthesis and metabolism of neurotrophic factors, thus causing the pathological changes of related nerve tissues involved in bladder function regulation [14, 15]. In the study, it is found that the knockout of Nrf2 gene alone affect the levels of oxidative products such as AGEs, and ROS in the bladder tissues of mice. Besides, the oxidative stress molecules in the bladder tissues of mice in T2DM group are increased than those in WT group and KO group. The oxidative stress products in KO-T2DM group are even obviously higher than those of the other three groups, and they are significantly different from T2DM group. Thus, the level of oxidative stress in the bladder tissues of T2DM mice is significantly increased, and the level of oxidative stress in the T2DM group is more serious than that in the T2DM group after Nrf2 knockout.

Compared with WT group and KO group, the expression of antioxidant stress molecule GSH and SOD in WT-T2DM group obviously decrease. Besides, the expression levels of GSH and SOD in KO-T2DM group are obviously lower than those in the other three groups. Therefore, the anti-oxidative stress ability of bladder tissue decreases obviously after the knockout of Nrf2 (Fig. 4).

In addition, the change of the important antioxidant molecules such as Quinone Oxidoreductase 1 (NQO1) and Heme Oxygenase 1 (HO-1) [16, 17] at the downstream of NRF2-ARE pathway is detected.

Nrf2 gene knockout alone does not affect the regulation of oxidative stress in the bladder of mice. The expression levels of NQO1 and HO-1 in the bladder tissues of WT-T2DM group are obviously lower than those of WT group and KO group. Besides, the expression levels of NQO1 and HO-1 in KO-T2DM group are significantly lower than those of the other three groups. According to the above results, Nrf2-ARE regulation pathway is impaired in T2DM mice; the expression and activity of downstream antioxidant protein are significantly decreased, while oxidative stress products are significantly increased. Furthermore, the oxidative stress regulation ability of bladder tissues in WT-T2DM group is impaired, while the oxidative stress metabolism in bladder tissues of KO-T2DM group becomes more seriously imbalanced (Fig. 4).

The oxidative stress caused by hyperglycemia is a vital cause of bladder tissue cell injury and apoptosis [18]. T2DM can increase MDA and AGEs in the body, activate aldose reductase and polyol pathway, and finally further increase ROS in the body [19]. Based on the existing studies, ROS can induce bax to enter mitochondria, release cytochrome C and activate caspase molecules to mediate cell apoptosis [20]. Moreover, ROS can degradate and down-regulate Bcl-2 protein through ubiquitin–proteasome pathway, and the expression of bax molecule is up-regulated to initiate downstream apoptosis pathway. As a result, it causes injury and apoptosis of smooth muscle cells, and ultimately bladder dysfunction [21, 22]. In the study, the expression levels of related apoptotic molecules in the bladder tissues of mice in each group are detected through western blotting and immunohistochemical staining. As seen from the findings, the expression of anti-apoptotic protein bcl-2 is down-regulated, while the expression of bax is up-regulated; the expression of caspase-3 is increased in the bladder tissues of mice in WT-T2DM group. The expression levels of these molecules regulating apoptosis are further increased in the bladder tissues of KO-T2DM group (Fig. 5). Therefore, oxidative stress can induce the apoptosis of bladder tissue cells by activating apoptosis, aggravate the injury and apoptosis of bladder tissue cells of diabetic mice after further inhibition of Nrf2 pathway, and speed up DBD.

Diabetic neuropathy is the potential etiological mechanism inducing DBD. Besides, the generation and metabolism disturbance of nerve growth factor (NGF) in diabetic pathology may be an important pathogenic factor inducing neurohistopathy. The pro-form of the nerve growth factor (pro-NGF) and NGF have different physiological functions. To be specific, Pro NGF has a high affinity for p75NTR receptor and promotes apoptotic signal transduction, while NGF has a high affinity for TrkANTR receptor and can activate nutritional signaling pathways in nerve cells [23]. The relatively stable expression levels of Pro NGF and NGF molecules can effectively maintain the normal physiological functions of nerve cells. If the expression of NGF significantly decreases in bladder tissue, it can result in nerve cell apoptosis, the decreased bladder sensation and incomplete bladder empty. The phenomenon is more common in patients with a long course of T2DM [24]. In addition, studies have shown that activating Nrf2 pathway can increase NGF expression to promote neurite growth of neurons after high glucose treatment [25]. Nrf2 in astrocytes produces antioxidant effects through glutamate receptors and intracellular Ca^2+^ signaling, and plays a neuroprotective role in surrounding neurons and synapses [26]. When anthocyanin is used to induce the expression of NRF2-ARE pathway in mice bladder, it can significantly increase the expression of NGF in the bladder tissue and improve the bladder dysfunction of T2DM mice [27]. In this study, the relative expression levels of pro-NGF and NGF in the bladder tissues of four groups of mice were detected through protein imprinting technology. According to the results, compared with WT group and KO group, the expression levels of pro-NGF in the bladder of T2DM group were significantly increased, while the NGF were significantly decreased. The NGF in KO-T2DM group was significantly lower than that in the other three groups, but the expression level of pro-NGF was obviously higher than that in the other three groups (Fig. 6A, B). The results show that the decrease of NGF expression in the bladder tissue of DM mice is related to the inhibition of Nrf2 molecule.

In conclusion, the successfully-established mouse model on Nrf2 knockout DM DBD mice proves that Nrf2 gene plays an important role in the process of DM DBD. To be specific, the down-regulated expression of Nrf2 gene can further aggravate the injury and apoptosis of diabetic mice bladder tissue cells, worsen oxidative stress injury process, and reduce cell oxidative stress resistance. Moreover, the down-regulation of Nrf2 gene can result in the formation disorder and down-regulation of NGF, and it is also the potential cause of bladder dysfunction.

## Data Availability

The data that support the findings of this study are available from the corresponding author upon reasonable request.
